# Impact of physiotherapy on orofacial manifestations of juvenile idiopathic arthritis

**DOI:** 10.1186/s12969-023-00900-0

**Published:** 2023-10-12

**Authors:** Stratos Vassis, Cory M. Resnick, Mette Nørgaard, Kathleen M. Strawn, Helle Grove, Beatrice Noeldeke, Troels Herlin, Thomas Klit Pedersen, Peter Bangsgaard Stoustrup

**Affiliations:** 1 Section of Orthodontics, Department of Dentistry and Oral Health, Aarhus University, Vennelyst Blvd. 9, Aarhus C, DK-8000 Denmark; 2 Department of Oral and Maxillofacial Surgery, Harvard Medical School, 188 Longwood Avenue, Boston, MA 02115 United States of America; 3 Department of Physiotherapy, Aarhus University Hospital, Skejby, Denmark, Palle Juul- Jensens Boulevard 103, Aarhus N, 8200 Denmark; 4 Department of Physical and Occupational Therapy, Boston Children’s Hospital, 300 Longwood Ave., Boston, MA 02115 United States of America; 5 Mårslet Fysioterapi & Traeningscenter, Hørretvej 16 C, Mårslet, 8320 Denmark; 6 Leibniz University Hannover, Welfengarten 1, 30167 Hannover, Germany; 7 Department of Clinical Medicine, Department of Paediatrics, Aarhus University, Palle Juul- Jensens Boulevard 103, Aarhus N, 8200 Denmark

**Keywords:** Juvenile idiopathic arthritis, Physiotherapy, Home exercises, Orofacial symptoms, TMJ arthritis

## Abstract

**Background:**

Physiotherapy appears as a promising therapy option for patients with Juvenile Idiopathic Arthritis (JIA) [1, 2], but the effects of physiotherapy and jaw exercises on JIA-related orofacial symptoms remain unknown [3]. The aim of this proof-of-concept study was to assess the impact of orofacial physiotherapy and home-exercise programs in patients with JIA and temporomandibular joint (TMJ) involvement.

**Methods:**

Twelve patients with JIA and TMJ involvement received a treatment of physiotherapy, complemented by prescribed home exercises spanning over eight weeks. Orofacial symptoms and dysfunction were monitored pre-treatment, during treatment, after treatment, and at a three-months follow-up.

**Results:**

Orofacial pain frequency and intensity significantly decreased during the course of the treatment (p = 0.009 and p = 0.006), with further reductions observed at the three-month follow-up (p = 0.007 and p = 0.002). During treatment, the mandibular function improved significantly in terms of maximal mouth opening capacity, laterotrusion, and protrusion.

**Conclusions:**

This proof-of-concept study shows favourable effects of physiotherapy and home excercises in the management of JIA-related orofacial symptoms and dysfunctions.

**Supplementary Information:**

The online version contains supplementary material available at 10.1186/s12969-023-00900-0.

## Background

Juvenile idiopathic arthritis (JIA) is the most common chronic rheumatic disease affecting children and adolescents and has an onset before the age of 16. It has a worldwide prevalence of 15–150 cases per 100,000 children and can cause symptoms such as joint pain, inflammation, damage, and functional limitation, leading to a lower quality of life [4–6]. The temporomandibular joint (TMJ) is frequently affected causing orofacial symptoms and dysfunctions, skeletal and dental deformities, and functional impairment [7, 8]. Recommendations for optimal management of JIA-related orofacial conditions include an interdisciplinary team consisting of pediatric rheumatologists, maxillo-facial surgeons, orthodontists, and physiotherapists combining pharmacological, surgical, and orthodontic treatments, as well as physiotherapy [3, 9].

Patients with JIA and TMJ involvement frequently require management of persistant orofacial pain and disability [10]. Management strategies include pain-coping, medication adjustment, use of orthopedic splints (stabilization splints), physiotherapy with combined home exercises, medication, intra-articular steroid injections, intra-articular lavage/arthrocentesis and/or arthroscopy, and open TMJ surgery/replacement [3]. While physiotherapy and home exercises present as potential therapeutic interventions for patients with JIA [1, 2], the effects of physiotherapy and exercises on orofacial symptoms associated with JIA remain undetermined [3].

More homogeneity in exercise and physiotherapy interventions are needed to draw robust conclusions on how physiotherapy and regular jaw exercises can alleviate JIA symptoms [1, 2].

Knowledge of the effect of physiotherapy targeting orofacial symptoms in patients with JIA is sparse. The purpose of this proof-of-concept study was to assess the effects of orofacial physiotherapy and a developed home-exercise program for patients with JIA and TMJ involvement. With this study, we hope to gain sufficient experience and evidence to proceed with randomization studies.

## Materials and methods

### Study cohort

Eligible patients were recruited from the Regional Craniofacial Clinic, Section of Orthodontics, Aarhus University, Denmark, and the Department of Plastic and Oral Surgery at Boston Children’s Hospital, MA, US. Inclusion criteria for enrollment were: (a) JIA diagnosis according to International League of Associations for Rheumatology (ILAR) criteria [11], (b) age over 10 years, (c) magnetic resonance imaging (MRI) and/or cone beam computed tomography (CBCT) findings of TMJ involvement, and (d) TMJ dysfunction and/or orofacial symptoms. The patients gave written consent to participate in this study and to publish the data in line with the rules of the Danish Health Authorities. The current study complies with the TMJaw consensus-based terminology for orofacial manifestations of JIA [9].

### Treatment and evaluation protocol

**Fig. 1:**
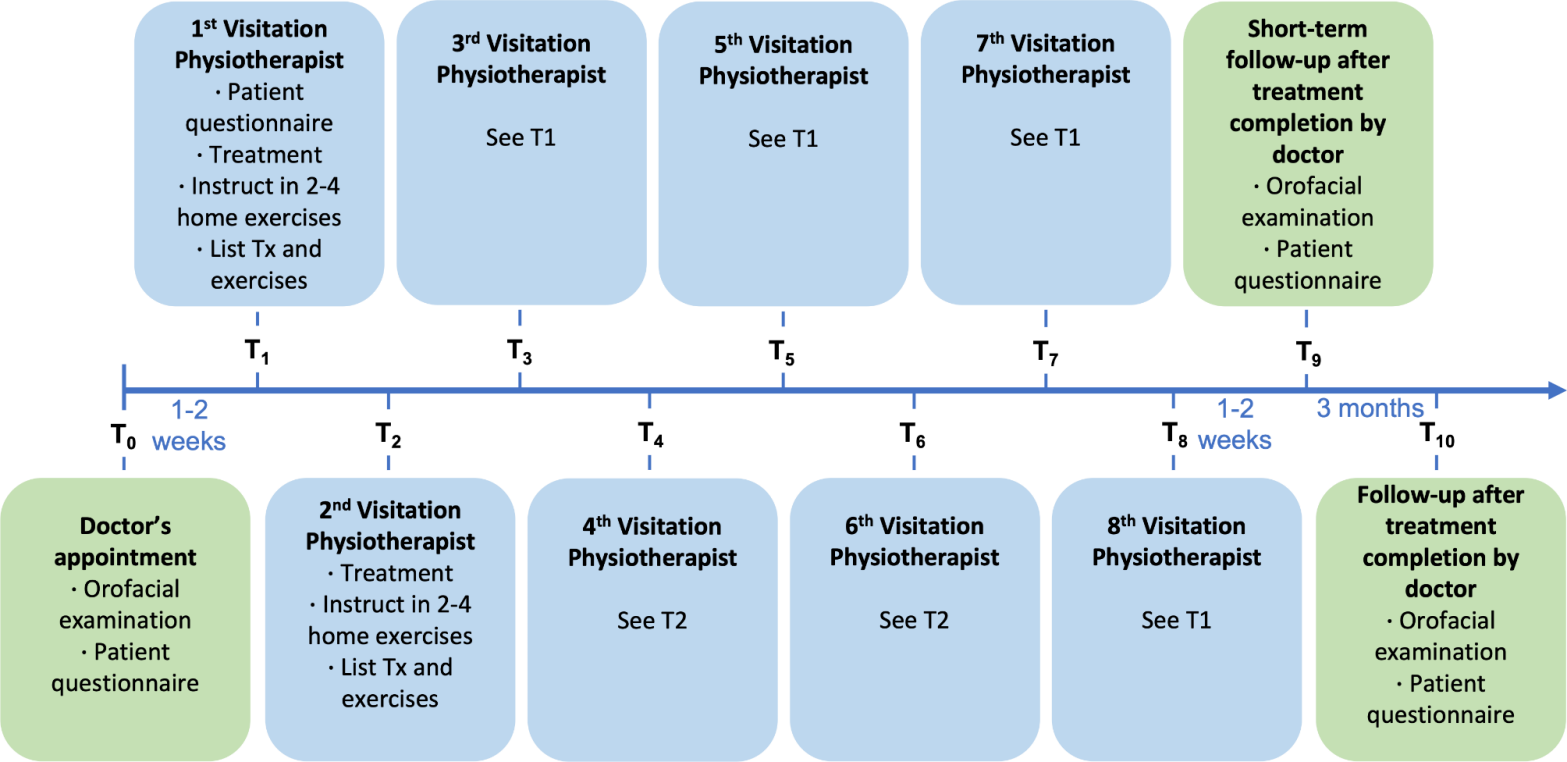
Outline Boston-Aarhus PT project protocol Note: Green: doctor’s appointments, blue: physiotherapist visitations. If not specified otherwise, time span is one week. Tx Treatment

A novel physiotherapy treatment protocol was defined by specialized physiotherapists for this study (Fig. 1). At T0 (pre-treatment), subjects underwent clinical examination utilizing a standardardized orofacial evaluation [12] (Appendix 1) and answered a validated 7-item questionnaire for the assessment of orofacial symptoms related to JIA [13] (Appendix 2). Subsequently, patients attended weekly physiotherapy sessions for eight weeks (T1 – T8). During each session, the physiotherapist performed treatment chosen from a list of treatment options and home exercises listed in Appendix 3 and Appendix 5 based on the physiotherapist’s clinical judgement and assessment of patient’s needs in compliance with the developed program. At T1, T3, T5, and T7, the 7-item symptom questionnaire was repeated. Both the symptom questionnaire and the standardized orofacial examination were repeated at two weeks after the last physiotherapy session (T9, “short-term follow-up”) and after three months (T10, “follow-up”).

### Treatment options

**Fig. 2:**
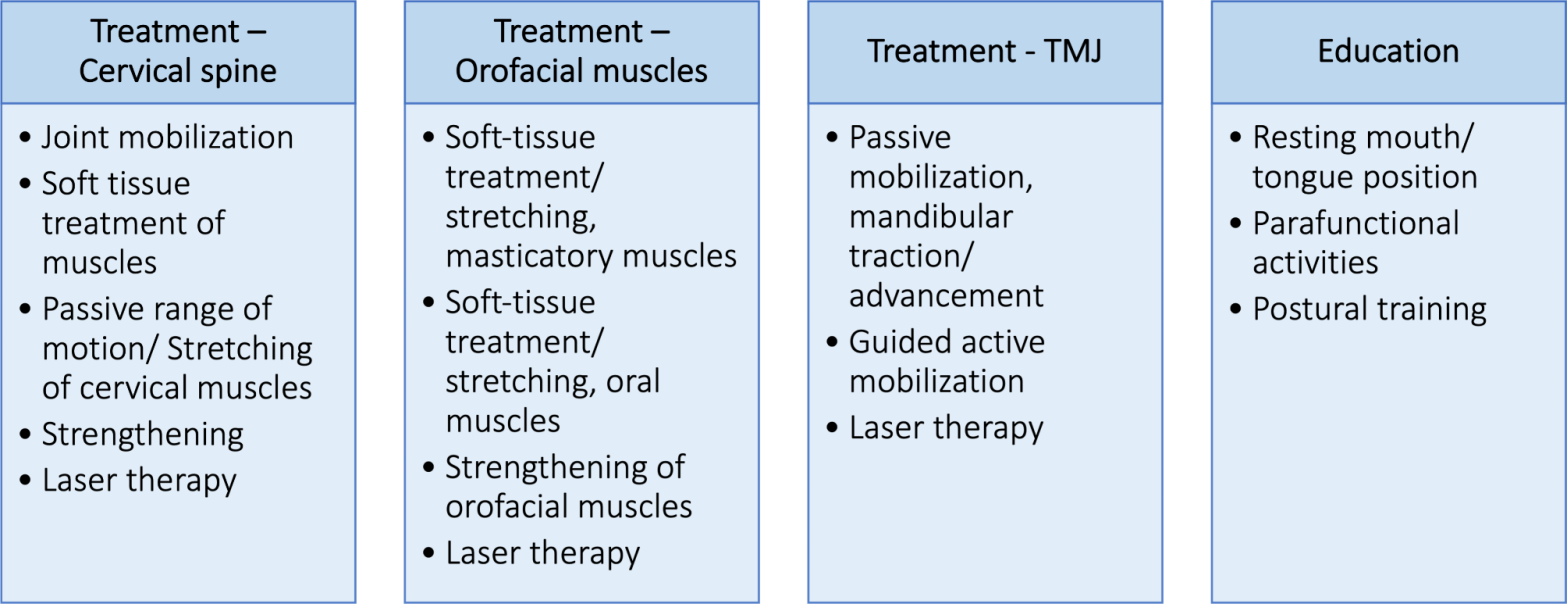
Physiotherapy treatment options

**Fig. 3:**
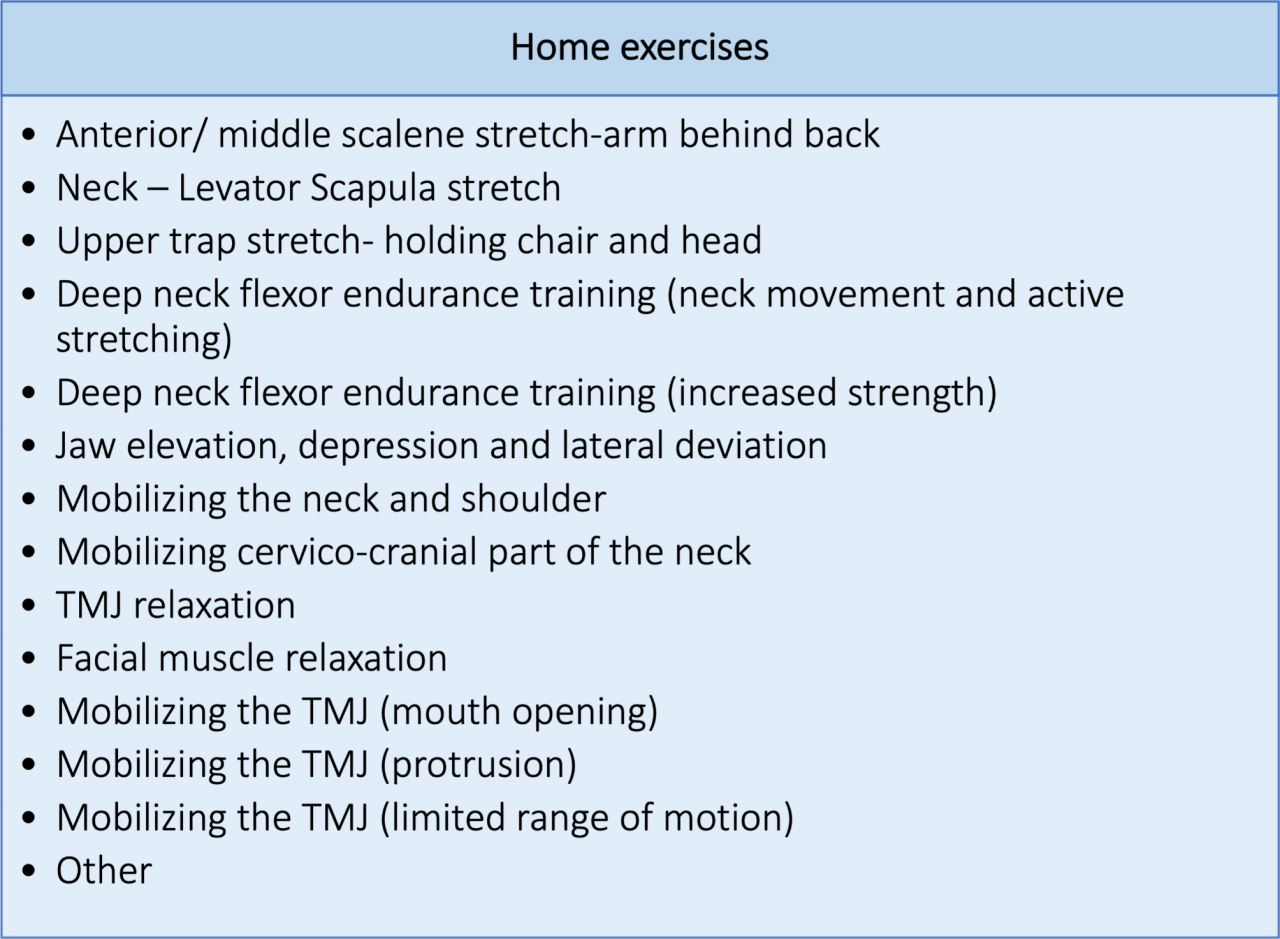
Home exercise options Note: ”Other” relates to supplementary exercises not mentioned in the exercise booklet

Therapeutic interventions were conducted by a physiotherapist who was instructed in the management of JIA-TMJ disease by a specialist physiotherapist. The treating physiotherapist selected from the treatment options as displayed in Fig. 2 and further explained in Appendix 3. Figure 3 visualizes the different home exercise options the physiotherapist could prescribe. To ensure correct execution, all patients were given written guidelines describing and illustrating each exercise in detail (Appendix 4).

### Outcome variables

The outcome variables include patient-reported orofacial symptom assessment documented through the questionnaire, orofacial dysfunction captured during clinical orofacial examinations, and self-reported patient compliance as described in Appendix 1 and Appendix 2.

### Statistical analysis

Descriptive statistics were used to display data. To test for significant differences in the outcome variables over time (T0-T10), the non-parametric Friedman test was applied, given the small sample size, non-normal distribution, and heterogeneity of variance. The Wilcoxon signed rank test was used for pairwise comparisons between outcome variables to test for differences between T0, T9, and T10. The statistical analysis was conducted in Stata 16.1 (Stata Corp. 2019. Stata Statistical Software: Release 16. College Station, TX: Stata Corp LLC.).

## Results

### Cohort description

Sixteen patients were enrolled across the two included centers. Of these, four were excluded due to interrupted course of management due to Covid-19 restrictions. Consequently, twelve patients were included in the statistical analysis.

The median age was 15 years [interquartile range 13–17 years] with total age range of 12–23 years. JIA baseline cohort information is displayed in Table 1. The most common medical treatment was biologics, received by six patients. Out of these patients, three received a combination of biologics and disease-modifying antirheumatic drugs (DMARDs). In total, non-steroidal anti-inflammatory drugs (NSAID) and DMARDS were prescribed to five patients each. One patient received TMJ intra-articular corticosteroids additional to NSAID and biologics. Out of the patients for which a contrast enhanced MRI was available, three patients displayed signs of acute TMJ inflammation, while for the other two no signs of acute TMJ inflammation were visible.

**Table 1:** Baseline cohort information

Cohort characteristics	n = 12
Females, number (percentage)	12 (100%)
Age, years (IQR, years)	15.6 (13–17)
JIA diagnosis, individual	
RF-positive polyarticular	5
Psoriatic	3
Oligoarticular persistent	2
Undifferentiated JIA	2
Disease activity	
None	3
Light to moderate, single joint	2
Acute	7
Medication^a^	
Biologics	6
NSAID	5
DMARDS	5
TMJ intra-articular corticosteroids	1

*a: Some patients received a combination of medication. Abbreviations: IQR interquartile range, JIA juvenile idiopathic arthritis, RF-positive polyarticular rheumatoid factor positive polyarticular, NA not available, NSAID non-steroidal anti-inflammatory drug, DMARDS disease-modifying antirheumatic drugs, TMJ temporomandibular joint*

### Physiotherapeutic interventions and home exercises

Most commonly, the physiotherapist treated the masticatory muscles by manual soft tissue/stretching (89.6%), strengthened the orofacial muscles (88.5%), and performed guided active TMJ mobilization (86.5%) (Table 2). The full overview of chosen treatment options at every visit are presented in the Appendix 6 (table A1).

**Table 2:** Performed physiotherapy exercises

Performed physiotherapy exercise	Share of patients receiving exercise (average over T1-T8) (in %)
**Cervical spine**	
Passive range of motion/Stretching of cervical muscles	75
Soft tissue treatment of muscles etc.	68.8
Strengthening	68.8
Joint mobilization	41.7
Laser therapy	0
**Orofacial muscles**	
Soft tissue Tx/Stretching (masticatory)	89.6
Strengthening of orofacial muscles	88.5
Soft tissue Tx/Stretching (oral)	78.1
Laser therapy	0
**TMJ**	
Guided active mobilization	86.5
Passive mobilization, mandibular traction/advancement	78.1
Laser therapy	21.9
**Education**	
Resting mouth/tongue position	78.1
Parafunctional activities	53.1
Postural training	28.1

### Home exercises

The physiotherapist prescribed facial muscle relaxation to 72.9% of the patients, TMJ mobilization (71.9%), jaw elevation, depression and lateral deviation (66.7%), and TMJ relaxation (63.5%) as exercises for the patients to do at home. Prescribed home exercises are displayed in Table 3; full overview of home exercises prescribed at every visit are included in table A2 in Appendix 6.

**Table 3:** Prescribed home exercises

Prescribed home exercises	Share of patients prescribed with exercise (average over T1-T8) (in %)
Facial muscle relaxation	72.9
Mobilizing the TMJ (protrusion)	71.9
Jaw elevation, depression, and lateral deviation	66.7
TMJ relaxation	63.5
Upper trap stretch-holding chair and head	60.4
Deep neck flexor endurance training (neck movement and active stretching)	46.9
Neck-Levator Scapula stretch	42.7
Deep neck flexor endurance training (increased strength)	38.5
Mobilizing the TMJ (mouth opening)	38.5
Mobilizing the TMJ (limited range of motion)	13.5
Anterior/Middle scalene stretch-arm behind back	7.1
Mobilizing neck and shoulder	5.2
Mobilizing cranial part of the neck	0

*Note: Average over time. One patient did not attend for the the last physiotherapy session at T8. Abbreviations: Tx treatment, TMJ temporomandibular joint*

### Compliance

**Fig. 4:**
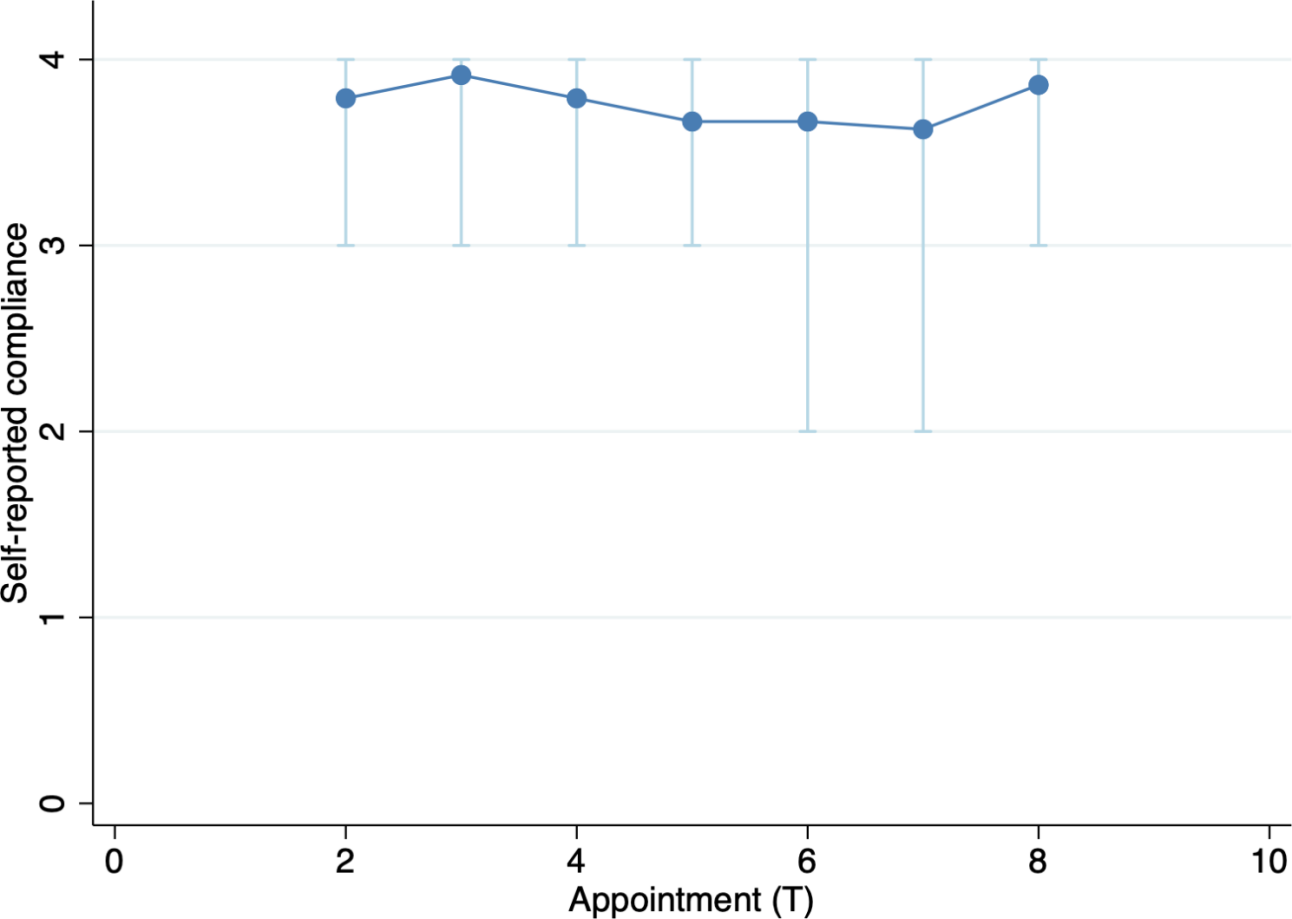
Self-reported compliance with home exercises assessed at physiotherapy visitation (T2-T8). Note: Light blue indicate variable range. Compliance was not reported at T1 since no home exercise was assigned at this point in time. Compliance measured as follows: 0 “Have not done the exercise at all” (0–10% compliance) 1 ”Not very often” (10–40% compliance) 2 “Somewhat done the exercises” (40–60% compliance) 3 “Almost done the exercises as instructed although missed a couple of times” (60–95% compliance) 4 “Done the exercises completely as instructed” (95–100% compliance)

Self-reported compliance with home exercises was high (Fig. 4). The majority (73.5%) of patients reported that they had “done the exercises completely as instructed”, and all subjects reported having at least “somewhat done the exercise” at all time points.

#### Orofacial symptoms

**Fig. 5:**
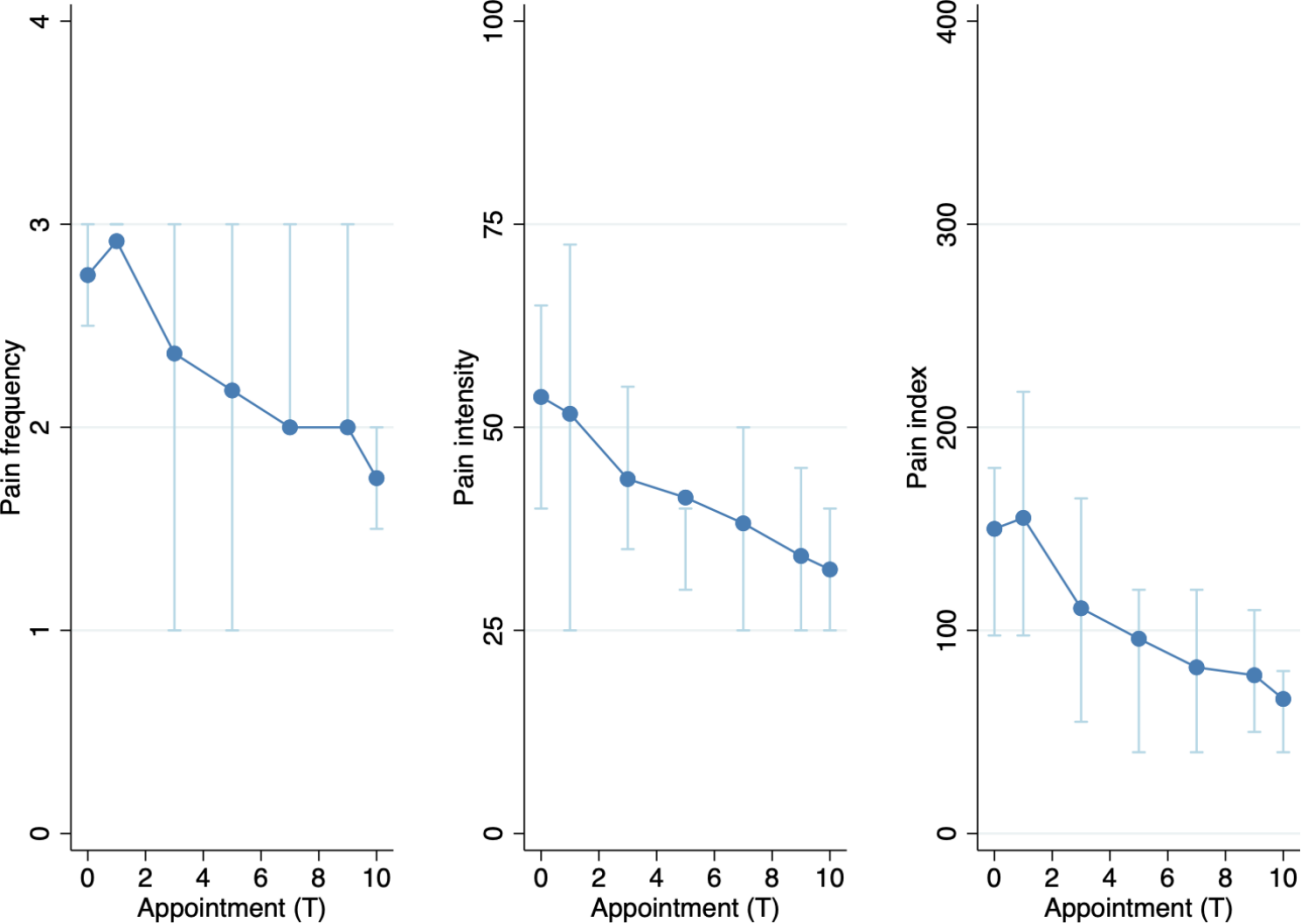
Pain intensity (in mm), pain frequency (0–4), pain index (pain intensity multiplied by pain frequency) at each appointment (T0-T10). Note: Light blue bars indicate interquartile range

Pain frequency, pain intensity, and the pain index significantly decreased during the course of treatment (p < 0.0002) (Fig. 5). At T0, 66.7% reported pain in the face or jaw several times per day (Table 4). By T10, only 8.3% experienced pain several times per day. Pain intensity decreased from 53.8 mm at T0 to 32.5 mm at T10 (p = 0.0002). Notably, both pain intensity and frequency decreased from T9-T10 after the weekly physiotherapy sessions had concluded.

**Table 4:** Orofacial symptoms

	T0 - Before treatment (SD)	T9 - Short-term follow-up (SD)	T10 - Follow-up (SD)	Diff. T9-T0/p-value*	Diff. T10-T0/p-value*	Diff. T10-T9/p-value*	Friedmann-test p-value* (T1,T2,T3,…T10)
Pain							
Pain frequency^a^	2.8 (0.8)	2.0 (0.9)	1.8 (0.8)	0.009	0.007	0.180	0.0002
Pain intensity (VAS 100 mm)	53.8 (17.3)	34.2 (17.9)	32.5 (16.0)	0.006	0.002	0.622	0.0002
Pain index (frequency x intensity)	150.0 (77.5)	77.9 (52.0)	66.3 (49.1)	0.005	0.003	0.15	0.0001

*Note: Intra-patient pariwise comparisons. Pain intensity was measured as mm on VAS* ^*a*^ *0: Never, 1: Less than once a week, 2: Several times a week, 3: Several times a day, 4: All the time*

#### TMJ and orofacial dysfunctions

The mouth opening capacity with and without pain, laterotrusion, and protrusion all improved between T0 and T9 (p < 0.054) (Table 5). Mouth opening ability and laterotrusion continued to improve after physiotherapy had been concluded. There was a non-significant trend toward reduction of crepitation during treatment. In contrast, translation was unchanged throughout the treatment timeline.

**Table 5:** TMJ and orofacial dysfunction

	T0 - Before treatment (SD)	T9 - Short-term follow-up (SD)	T10 - Follow-up (SD)	Diff. T9-T0/p-value*	Diff. T10-T0/p-value*	Diff. T10- T9/p-value*
Function						
Mouth opening without pain (mm)	36.0 (8.3)	40.4 (7.5)	43.0 (8.7)	0.054	0.045	0.084
Mouth opening with pain (mm)	41.4 (6.5)	47.6 (5.0)	48.8 (7.0)	0.003	0.006	0.679
Laterotrusion (average) (mm)	7.8 (1.3)	9.1 (1.2)	9.5 (1.5)	0.006	0.005	0.968
Protrusion (mm)	6.8 (1.8)	8.3 (1.7)	7.9 (1.4)	0.006	0.027	0.303
TMJ with crepitation^a^	0.25	0.18	0.17	0.157	0.317	1.000
TMJ with decreased translation (%)	25%	25%	25%	1.000	1.000	1.000
Temporalis tenderness^b^	0.3	0.2	0.3	0.297	1.000	0.297
Masseter tenderness^b^	0.8	0.3	0.6	0.009	0.084	0.027

^a^ 0: not present, 1: light crepitation, 2: servere crepitation; ^b^ 0: no tenderness, 1: moderate tenderness, 2: severe tenderness

Temporalis and masseter tenderness decreased during active treatment (p = 0.297 and p = 0.009). By T10, both temporalis and masseter tenderness rebounded, but masseter tenderdness remained below T0 levels.

## Discussion

In this proof-of-concept study, we investigated how orofacial physiotherapy and home-exercise programs affected patients with JIA and TMJ involvement. The findings demonstrated that orofacial pain frequency and intensity significantly decreased throughout the treatment period, with additional reductions observed at the three-month follow-up. During treatment, the mandibular function enhanced significantly as evidenced by improved maximal mouth opening capacity, laterotrusion, and protrusion.

Physiotherapy and therapeutic exercises are currently utilized to reduce facial pain, enhance TMJ function, increase active and passive mouth opening capacity in temporomandibular disorders [3, 12, 14–16]. However, little is known about the impact of physiotherapy on JIA-related orofacial manifestations. The 2023 interdisciplinary consensus-based recommendations on the management of orofacial manifestations of JIA propose various orofacial pain management strategies, such as implementing a stabilizing splint, prescribing physiotherapy and/or medication, and informing about further pain-avoidance measures [3, 17]. The combination of splint therapy and physiotherapy is considered a safe and reversible option [3]. However, these recommendations are supported solely by empirical evidence and hence require further investigation [3]. Furthermore, orofacial exercise concerning patients with JIA are not described. The main objective of our proof-of-concept study was to evaluate the effectiveness of orofacial physiotherapy and suggest a home exercise program for patients with JIA and orofacial symptoms.

According to our results, orofacial pain frequency and intensity significantly decreased over the course of the treatment. Tabeian et al. suggested that oral physiotherapy or well-functioning orthodontic splints, which apply pressure on the affected TMJ, reduce the catabolic impact of tumor necrosis factor-alpha (TNF-α) on the TMJ [17, 18]. Also, von Bremen et al. have shown in an experimental model that functional joint loading reduces inflammation and positively affects condylar growth at a histological level [19]. In our study, pain did not recur by three months after active treatment cessation. This sustained effect could be attributed to a change in the inflammatory milieu induced by physiotherapy and/or prescribed home exercises.

Our results indicate that physiotherapy also improves mouth opening capability, laterotrusion, and protrusion. These findings are in line with other studies [20–22]. Crepitation and translation did not significantly benefit by physiotherapy in our sample. Although physiotherapy and home exercises seems to improve TMJ dysfunction, this indicates that full TMJ recovery may not be achieved in patients with JIA and TMJ deformity. This is expected if arthritis related structural alterations or degeneration of the TMJ are present. The sustainability of the beneficial effects of therapy also remains unclear, as some rebound tenderness of the masseter and temporalis muscles was observed after treatment cessation, though the pre-treatment level was not reached over our three month follow-up timeline (for masseter).

In our study, the most common physiotherapy interventions were treating soft tissue/stretching for masticatory muscles, strengthening of orofacial muscles, anterior/caudal traction and guided active TMJ mobilization. Shimada et al. found that mobilization therapy including manual therapy and passive jaw mobilization with oral appliances improved TMJ functioning for patients with painful TMJ disorders [23]. The most frequently prescribed home exercises in this study comprised facial muscle relaxation, TMJ mobilization, jaw elevation, depression and lateral deviation, and TMJ relaxation. In Shimada et al.‘s research, voluntary jaw exercises served as a useful complement to physiotherapy in reducing myalgia and arthralgia [23]. Mienna et al. hypothesized that individualized exercises in the rehabilitation process of TMJ dysfunction increase the confidence in patients with chronic TMJ pain [24]. This could explain the high degree of compliance with home exercises. Our results extend existing literature by demonstrating that the positive affects of physiotherapy and high compliance can also be achieved in children and adolescents with JIA and TMJ-involvement.

This study is limited by the small sample size. Also, the follow-up time is relatively short and the potential for additional relapse of the therapy benefits beyond the study timeline cannot be assessed. One patient suffered from a sinus infection at T0, but the main conclusions remain robust after removing this patient from the analysis. As compliance is self-reported, it might be subject to response bias. Additionally, benefical changes in our sample may reflect natural fluctuation of the disease and orofacial symptoms rather than treatment effect. The study did not assess the interference of medication with the treatment. Furthermore, it remains unclear whether the positive outcomes result from physiotherapy or home exercises or can only be obtained through their combination. The strengths of the study are the prospective design, the recommended exercise options and standardized examination form. Further research should apply the implemented treatment plan to a larger sample of patients with longer follow-up periods in randomized controlled set-ups. Furthermore, future studies should investigate how medication changes and systemic inflammation mediate the treatment effect of physiotherapy.

## Conclusions

The results of this proof-of-concept study suggest a beneficial effect of physiotherapy and home exercises on JIA-related orofacial symptoms and dysfunctions. These findings support a place for physiotherapy and home excercises in the management of orofacial manifestations of JIA.

Tables.

### Electronic supplementary material

Below is the link to the electronic supplementary material.

Supplementary Material 1

## Data Availability

The questionnaires and the list of treatment options and home exercises are included in the Appendices. The patients clinical data are not publicy available.

## List of Abbreviations

CBCT: Cone beam computed tomography
DMARDs: Disease-modifying antirheumatic drugs
ILAR: International League of Associations for Rheumatology
JIA: Juvenile idiopathic arthritis
MRI: Magnetic resonance imaging
NSAID: Non-steroidal anti-inflammatory drugs
TMJ: Temporomandibular joint
TNF-α: Tumor necrosis factor-alpha
