# Supplementary material for: Impact of physiotherapy on orofacial manifestations of juvenile idiopathic arthritis
Source: Pediatr Rheumatol Online J. 2023 Oct 12;21:116. doi: 10.1186/s12969-023-00900-0 (PMC10571390; doi:10.1186/s12969-023-00900-0)
Supplement: Supplementary file 1 — Supplementary Material 1 [file 12969_2023_900_MOESM1_ESM.docx]

***Appendix 1: General information and orofacial examination protocol***

***Appendix 2: euroTMJoint-7-item questionnaire***

***Appendix 3: Clinical guidelines***

***Appendix 4: Home exercise program***

***Appendix 5: Questionnaire - treatment and home exercises***

***Appendix 6: Further results***

Table A1: Physiotherapy exercises over time. Full overview.

| **Physiotherapy exercise** | **T1 (n=12)** | **T2** | **T3** | **T4** | **T5** | **T6** | **T7** | **T8** |
| --- | --- | --- | --- | --- | --- | --- | --- | --- |
| **Cervical spine** |  |  |  |  |  |  |  |  |
| Joint mobilization | 7 (58.3%) | 8 (66.7%) | 5 (41.7%) | 4 (33.3%) | 3 (25.0%) | 5 (41.7%) | 4 (33.3%) | 4 (33.3%) |
| Soft tissue treatment of muscles etc. | 9 (75.0%) | 8 (66.7%) | 9 (75.0%) | 9 (75.0%) | 8 (66.7%) | 9 (75.0%) | 7 (58.3%) | 7 (58.3%) |
| Passive range of motion/Stretching of cervical muscles | 11 (91.7%) | 10 (83.3%) | 10 (83.3%) | 11 (91.7%) | 8 (66.7%) | 9 (75.0%) | 6 (50.0%) | 7 (58.3%) |
| Strengthening | 9 (75.0%) | 10 (83.3%) | 9 (75.0%) | 8 (66.7%) | 9 (75.0%) | 8 (66.7%) | 7 (58.3%) | 6 (50.0%) |
| Laser therapy | 0 (0%) | 0 (0%) | 0 (0%) | 0 (0%) | 0 (0%) | 0 (0%) | 0 (0%) | 0 (0%) |
| **Orofacial muscles** |  |  |  |  |  |  |  |  |
| Soft tissue Tx/Stretching.  **Masticatory muscles** | 12 (100%) | 10 (83.3%) | 11 (91.7%) | 11 (91.7%) | 10 (83.3%) | 11 (91.7%) | 11 (91.7%) | 10 (83.3%) |
| Soft tissue Tx/Stretching  **Oral muscles** | 9 (75.0%) | 10 (83.3%) | 10 (83.3%) | 10 (83.3%) | 9 (75.0%) | 9 (75.0%) | 9 (75.0%) | 9 (75.0%) |
| Strengthening of orofacial muscles | 8 (66.7%) | 10 (83.3%) | 10 (83.3%) | 12 (100%) | 12 (100%) | 11 (91.7%) | 12 (100%) | 10 (83.3%) |
| Laser therapy | 0 (0%) | 0 (0%) | 0 (0%) | 0 (0%) | 0 (0%) | 0 (0%) | 0 (0%) | 0 (0%) |
| **TMJ** |  |  |  |  |  |  |  |  |
| Passive mobilization, mandibular traction/advancement | 11 (91.7%) | 0 (0%) | 11 (91.7%) | 11 (91.7%) | 9 (75.0%) | 11 (91.7%) | 11 (91.7%) | 11 (91.7%) |
| Guided active mobilization | 10 (83.3%) | 10 (83.3%) | 11 (91.7%) | 11 (91.7%) | 9 (75.0%) | 10 (83.3%) | 11 (91.7%) | 11 (91.7%) |
| Laser therapy | 0 (0%) | 0 (0%) | 0 (0%) | 0 (0%) | 0 (0%) | 0 (0%) | 0 (0%) | 0 (0%) |
| **Education** |  |  |  |  |  |  |  |  |
| Resting mouth/tongue position | 11 (91.7%) | 11 (91.7%) | 10 (83.3%) | 10 (83.3%) | 9 (75.0%) | 8 (66.7%) | 7 (58.3%) | 9 (75.0%) |
| Parafunctional activities | 8 (66.7%) | 7 (58.3%) | 6 (50.0%) | 7 (58.3%) | 6 (50.0%) | 6 (50.0%) | 4 (33.3%) | 7 (58.3%) |
| Postural training | 3 (25.0%) | 3 (25.0%) | 5 (41.7%) | 5 (41.7%) | 3 (25.0%) | 3 (25.0%) | 2 (16.7%) | 3 (25.0%) |

*Note: Tx treatment, TMJ temporomandibular joint.*

Table A2: Home exercises over time. Full overview.

| **Home exercise** | **T1 (n=12)** | **T2** | **T3** | **T4** | **T5** | **T6** | **T7** | **T8** |
| --- | --- | --- | --- | --- | --- | --- | --- | --- |
| Anterior/Middle scalene stretch-arm behind back | 0 (0%) | 1 (8.3%) | 1 (8.3%) | 1 (8.3%) | 1 (8.3%) | 1 (8.3%) | 2 (16.7) | 0 (0%) |
| Neck-Levator Scapula stretch | 3 (25.0%) | 5 (41.7%) | 6 (50.0%) | 6 (50.0%) | 6 (50.0%) | 6 (50.0%) | 4 (33.3%) | 5 (41.7%) |
| Upper Trap stretch-holding chair and head | 7 (58.3%) | 7 (58.3%) | 7 (58.3%) | 8 (66.7%) | 8 (66.7%) | 8 (66.7%) | 7 (58.3%) | 6 (50.0%) |
| Deep neck flexor endurance training (neck movement and active stretching) | 6 (50.0%) | 6 (50.0%) | 7 (58.3%) | 6 (50.0%) | 6 (50.0%) | 6 (50.0%) | 5 (41.7%) | 3 (25.0%) |
| Deep neck flexor endurance training (increased strength) | 5 (41.7%) | 5 (41.7%) | 5 (41.7%) | 5 (41.7%) | 5 (41.7%) | 5 (41.7%) | 4 (33.3%) | 3 (25.0%) |
| Jaw elevation, depression, and lateral deviation | 1 (8.3%) | 7 (58.3%) | 7 (58.3%) | 10 (83.3%) | 10 (83.3%) | 10 (83.3%) | 10 (83.3%) | 9 (75.0%) |
| Mobilizing neck and shoulder | 2 (16.7) | 0 (0%) | 0 (0%) | 0 (0%) | 1 (8.3%) | 1 (8.3%) | 0 (0%) | 1 (8.3%) |
| Mobilizing cranial part of the neck | 0 (0%) | 0 (0%) | 0 (0%) | 0 (0%) | 0 (0%) | 0 (0%) | 0 (0%) | 0 (0%) |
| TMJ relaxation | 7 (58.3%) | 7 (58.3%) | 7 (58.3%) | 8 (66.7%) | 9 (75.0%) | 9 (75.0%) | 8 (66.7%) | 6 (50.0%) |
| Facial muscle relaxation | 8 (66.7%) | 9 (75.0%) | 9 (75.0%) | 9 (75.0%) | 9 (75.0%) | 9 (75.0%) | 9 (75.0%) | 8 (66.7%) |
| Mobilizing the TMJ (mouth opening) | 2 (16.7%) | 4 (33.3%) | 5 (41.7%) | 5 (41.7%) | 5 (41.7%) | 6 (50.0%) | 6 (50.0%) | 4 (33.3%) |
| Mobilizing the TMJ (protrusion) | 9  (75.0%) | 2 (16.7) | 10 (83.3%) | 10 (83.3%) | 10 (83.3%) | 10 (83.3%) | 10 (83.3%) | 8 (66.7%) |
| Mobilizing the TMJ (limited range of motion) | 1 (8.3%) | 2 (16.7%) | 2 (16.7%) | 2 (16.7%) | 2 (16.7%) | 2 (16.7%) | 1 (8.3%) | 1 (8.3%) |

*Note: TMJ temporomandibular joint.*
